# Modeling microbial survival in buildup biofilm for complex medical devices

**DOI:** 10.1186/1471-2334-9-56

**Published:** 2009-05-08

**Authors:** Michelle J Alfa, Rosemarie Howie

**Affiliations:** 1 Department of Medical Microbiology, University of Manitoba, Winnipeg, MB R3T 2N2, Canada

## Abstract

**Background:**

Flexible endoscopes undergo repeated rounds of patient-use and reprocessing. Some evidence indicates that there is an accumulation or build-up of organic material that occurs over time in endoscope channels. This "buildup biofilm" (BBF) develops as a result of cyclical exposure to wet and dry phases during usage and reprocessing. This study investigated whether the BBF matrix represents a greater challenge to disinfectant efficacy and microbial eradication than traditional biofilm (TBF), which forms when a surface is constantly bathed in fluid.

**Methods:**

Using the MBEC (Minimum Biofilm Eradication Concentration) system, a unique modelling approach was developed to evaluate microbial survival in BBF formed by repetitive cycles of drying, disinfectant exposure and re-exposure to the test organism. This model mimics the cumulative effect of the reprocessing protocol on flexible endoscopes. Glutaraldehyde (GLUT) and accelerated hydrogen peroxide (AHP) were evaluated to assess the killing of microbes in TBF and BBF.

**Results:**

The data showed that the combination of an organic matrix and aldehyde disinfection quickly produced a protective BBF that facilitated high levels of organism survival. In cross-linked BBF formed under high nutrient conditions the maximum colony forming units (CFU) reached ~6 Log_10 _CFU/peg. However, if an oxidizing agent was used for disinfection and if organic levels were kept low, organism survival did not occur. A key finding was that once established, the microbial load of BBF formed by GLUT exposure had a faster rate of accumulation than in TBF. The rate of biofilm survival post high-level disinfection (HLD) determined by the maximum Log_10_CFU/initial Log_10_CFU for *E. faecalis *and *P. aeruginosa *in BBF was 10 and 8.6 respectively; significantly different compared to a survival rate in TBF of ~2 for each organism. Data from indirect outgrowth testing demonstrated for the first time that there is organism survival in the matrix. Both TBF and BBF had surviving organisms when GLUT was used. For AHP survival was seen less frequently in BBF than in TBF.

**Conclusion:**

This BBF model demonstrated for the first time that survival of a wide range of microorganisms does occur in BBF, with significantly more rapid outgrowth compared to TBF. This is most pronounced when GLUT is used compared to AHP. The data supports the need for meticulous cleaning of reprocessed endoscopes since the presence of organic material and microorganisms prevents effective disinfection when GLUT and AHP are used. However, cross-linking agents like GLUT are not as effective when there is BBF. The data from the MBEC model of BBF suggest that for flexible endoscopes that are repeatedly used and reprocessed, the assurance of effective high-level disinfection may decrease if BBF develops within the channels.

## Background

Flexible endoscopes are widely and increasingly used for diagnostic and therapeutic procedures, e.g. colonoscopy is the gold standard for colon cancer screening. However, these reusable medical devices pose a unique challenge to infection control. They are complex in design with multiple long, narrow, cross-connected lumens. Due to the material composition, the majority of flexible endoscopes cannot be steam sterilized. Contact with mucosal surfaces in the body necessitates a minimum of HLD. Flexible endoscopes are repeatedly reprocessed up to a thousand times per year. Reprocessing involves cleaning (generally with an enzymatic detergent), HLD (most often with a liquid chemical disinfectant), rinsing, drying and storage.

Spach et al. [1] described nosocomial transmission of microorganisms by endoscopes as a result of scope contamination from the patient and/or the inanimate environment (e.g. irrigating solutions, automated endoscope reprocessing devices). If organisms from these sources survive the reprocessing steps they could then lead to subsequent patient infection. Survival of the reprocessing stage may result if the HLD concentration is inadequate (e.g. dilution of HLD), if cleaning is inadequate or if the scope is contaminated post-processing. Although the reported incidence of infection transmission is very low, this has been attributed in part to unreported or unrecognized (i.e. infection occurs but the link with a recent endoscopic procedure is not recognized because the physician dealing with the infection is not the physician who performed the endoscopy) transmission. It is expected that strict adherence to recommended reprocessing protocols will effectively eliminate all bioburden from the endoscope (i.e. microbes and organic matter from patient secretions). Most reports of infection transmission have been associated with breeches in the reprocessing protocol but as noted by Vickery et al. [2] "These studies suggest that human error is a major issue, but we and others have found > 2% bacterial contamination rate of patient-ready endoscopes despite good adherence to infection control guidelines." Nelson indicated that in reprocessed flexible endoscopes, residual organic matter and biofilm is likely a result of "multiple cleaning and disinfection cycles over the life of the instrument [3]." Furthermore, there is evidence of microbial survival in patient-used scopes [4,5] including: *Mycobacterium tuberculosis *survival of multiple patient-use/reprocessing cycles for 17 days; [6]*Hepatitis C virus *(HCV) infection of three successive patients within a few days in spite of reprocessing; [7] and *Pseudomonas *contaminated scopes and infection of 100 out of 410 patients, attributable to a scope design flaw [8,9]. Biofilm formation in flexible endoscopes has also been shown by SEM or suggested by various researchers, [3,2,10,11] with implications in facilitating infection transmission [11,12]. Despite these findings there are no prospective published reports in the medical literature directly linking an endoscope, cleaned in accordance with current reprocessing standards and guidelines and not defective in design, to transmission of an infectious agent (biofilm associated or not) from one patient to another patient or from the environment to the patient.

Traditional biofilm (TBF) forms on a surface continually bathed in fluid and exposed to microorganisms (e.g. indwelling lines (catheter, IV) and medical implants). Development of traditional biofilm in endoscopes is thought to be associated with residual moisture in remaining channels [11,2] that likely originated from water sources, e.g. in AERs and moisture in channels involving such organisms as *Mycobacterium spp*, *Legionella spp*, and *Pseudomonas aeruginosa*. However, as reviewed by Pajkos et al. [11] the gradual build up of material over repeated use in reprocessed flexible endoscopes forms by a very different kinetic background. The initial stages of formation (surface conditioning from patient secretions, microbial attachment, growth and colonization) are similar to TBF. However medical devices such as gastrointestinal (GI) endoscopes are used repeatedly in a day, with cyclic exposure to high levels of microbes due to contact with the mucosal surface of the gut. In addition, each use-reprocessing cycle involves scope exposure to hydrated phases (post patient, cleaning, and disinfection) as well as a dry phase (during storage). Repeated use over time can facilitate a buildup biofilm formation (BBF) consisting of layers of dried organic material with embedded microorganisms. Pajkos et al. [11] reported that new endoscopes do not have soil or biofilm in their channels but that 12 or 13 endoscopes from 13 different hospitals did have accumulations detected by SEM. These SEM observations support the need to model the buildup over repeated use as it does appear that patient-used endoscopes have accumulations over time. We hypothesize that the resultant buildup biofilm (BBF) would have a unique composition, microbial proliferation, biofilm formation and survival characteristics compared to TBF.

Little is known regarding the progressive buildup of residual patient material and organisms within the channels of flexible endoscopes that are repeated reprocessed throughout the life of the device. To accurately study microbial survival within BBF and potential microbial transmission, a novel model of BBF was developed that replicates the reprocessing cycles and closely reflects the type of buildup biofilm that develops in medical devices that are reprocessed repeatedly. The BBF model was compared to TBF using the MBEC peg model [13,14] in the presence of high or low levels of organic material. The efficacy of microbial killing for two commonly used high-level disinfectants (glutaraldehyde, a cross-linking agent, and accelerated hydrogen peroxide, an oxidizing agent) was compared in these BBF and TBF models.

## Methods

### Microorganisms and culture

Test organisms in this research project were a representative range of microorganisms that could be associated with contamination of complex medical devices and/or healthcare environments and included: *Pseudomonas aeruginosa *ATCC15442, *Enterococcus faecalis *ATCC 29212, and *Candida albicans *ATCC 14053. Bacterial organisms were passaged on tryptic soy agar (TSA) (Oxoid, Toronto, Canada) medium at 35°C aerobically for 24 to 48 hours. Fungal organisms were sub-cultured on TSA supplemented with 5% whole sheep blood (BA) (Oxoid, Toronto, Canada) and incubated at 30°C aerobically for 72 hours. Stock cultures were maintained in skim milk at -70°C. All bacterial cultures were subcultured three times before experimentation.

### Test Disinfectants

Glutaraldehyde (Metricide™, Sybron Canada, Oakville, Canada) at a stock concentration of 2.6% (w/v) was tested for HLD as per APIC's currently accepted practice of 20 minutes exposure at room temperature [15,12]. The APIC recommendation for HLD using Glutaraldehyde differs from the manufacturer's recommended exposure time of 45 minutes at 25°C and is based on the expectation that the flexible endoscopes have been adequately cleaned prior to HLD. Accelerated hydrogen peroxide (PerCept, Virox, Mississauga, Canada) was evaluated at a 7% v/v for HLD concentration and tested as per manufacturer's recommendations (i.e. 20 minutes exposure at room temperature). Any dilution of disinfectants was done using sterile tap water, prepared immediately before each test and was not reused.

### Test soil

To provide an organic challenge that mimicked medical device exposure in the body, an artificial test soil (ATS) was used in all biofilm studies. ATS is composed of protein, carbohydrate, endotoxin and hemoglobin at worst-case levels as detected in patient-used flexible endoscopes [16,17]. The test soil without organic content was sterile tap water (sTAP). To represent the endoscope reprocessing conditions, an enzymatic detergent (Pentazyme (Case Medical Inc., Ridgefield, New Jersey, USA)) was used as a source of nutrition (feed) during biofilm formation.

### Traditional Biofilm (TBF) MBEC model

Traditional biofilm was formed in the MBEC system (Innovotech Ltd, Calgary, Alberta) under sterile conditions in a Class II B3 biosafety cabinet. Biofilm formation (and related controls) were established on and recovered from the pegs based on the manufacturer's recommendations and methods of Ceri et al. [13] and Harrison et al. [14]. The ATS was used for inoculation of pegs and for feeding. Pegs were exposed to ATS and organisms for 24 hours at RT to establish biofilm formation on all pegs. After 24 hours, the MBEC pegs were rinsed and all pegs were fed on a daily basis with ATS or tap water for 30 days. Pegs were removed and assayed at various intervals to monitor biofilm progression.

### Biofilm recovery from MBEC pegs

For biofilm recovery, a 96-well microtitre plate was prepared by adding 200 uL of sPBS per well. Alternately, when small numbers of pegs were recovered, rinsing took place in 500 uL sPBS per sterile microcentrifuge tube for each removed peg. Individual pegs were broken off the lid by placing a sterile haemostat at the lid/peg interface and breaking the peg off. Pegs were briefly rinsed in sPBS, 3 times for 20 seconds, to remove nonadherent material.

The rinsed pegs were transferred to recovery tubes containing sPBS in either a volume of 500 uL in a microfuge tube or in 1 mL in a 2 mL snap-cap test tube (Simport, Quebec, Canada). Tubes were shaken on a shaker table at high speed for 2 min, sonicated at 50/60 Hertz using a Bransonic 1200 Ultrasonic cleaner (Branson Canada, Pickering ON) for 5 minutes and vortexed for 1 minute. This recovery method has been recommended for MBEC pegs [13,14].

### Viability assay

Quantitative assessment (viable counts) of recovered viable biofilm bacteria was achieved using serial 1:10 dilutions in sPBS in conjunction with the spread plate technique on TSA or BA. The limit of detection for the viability assay was 10 CFU/peg.

### Buildup biofilm (BBF) model development

#### The BBF model

To model the buildup of material in a way that mimicked patient-used flexible endoscopes over time the MBEC pegs were exposed to repetitive cycles of treatments that were representative of the endoscope reprocessing protocol. This modelling approach was designed to evaluate microbial survivability in BBF formed by treatments that were repetitive cycles of drying, disinfectant exposure and re-exposure to the test organism.

Treatment cycles of 2 days mimicked overnight reprocessing conditions. Inoculated pegs were fed with nutrient media or HLD treatment post drying on alternating days. All test sampling was done immediately pre- and post-treatment cycle on triplicate sample pegs.

The MBEC pegs were inoculated using ATS for all experiments. The media used to feed the biofilm growth was ATS (representing a high organic matrix), sterile tap water (representing a low organic matrix, as found in scope rinsing), or an enzymatic detergent (representing a moderate organic matrix, as found in scope cleaning). For all treatments, drying was done ON (12 to 18 hours) at RT in a biosafety cabinet. HLD was achieved using glutaraldehyde and accelerated hydrogen peroxide (for 20 minute exposure times at RT as per manufacturer's recommendations). Following disinfectant exposure, pegs were neutralized using 10% FBS in TSB (for 10 minutes) to eliminate the possibility of disinfectant carryover to the recovery medium.

#### The Cyclic BBF model

To more closely resemble environments in the reprocessing scheme, biofilm growth was evaluated in enzymatic detergent (as used in the cleaning process) compared to sTAP (similar to endoscope rinsing stages). As well repetitive re-exposure to bioburden (as would be encountered for each new endoscopic procedure) finalized the cycle. The sample pegs were removed before and after HLD and evaluated for microbial survival.

#### BBF controls

Recoverable TBF bioburden was used as a positive control to define the CFU per peg for continuously formed biofilm. The TBF control was formed in MBEC plates at the same time, with the same inoculum samples, supplied with the same nutrient media lot, and with sample pegs removed and challenged with the same disinfectant lot and working solution at the same specified test times and conditions as for BBF. All test results were from a minimum of 9 replicates resulting from a minimum of triplicate replications from 3 separate trials.

To monitor the possibility of contamination or cross-contamination due to the cycling procedure, negative pegs (exposed to medium only) were located throughout the plate in sufficient numbers (resulting in a minimum of 3 negative pegs per test cycle).

#### Outgrowth testing

Following HLD, sample pegs were aseptically transferred into 10% FBS in TBS in sterile tubes that remained unopened at 35°C for 5 days. These tubes were subjected to the standard elution protocol (shaking for 2 minutes, sonication for 5 minutes, vortexing for 1 minute) however the tubes remained closed. The tubes with pegs were re-incubated for 25 days at 35°C. Turbidity indicated positive growth and if no turbidity was seen, the broth was still subbed to TSA or BA.

Concurrent to removing test pegs for the indirect qualitative outgrowth test, replicate sample pegs were removed and directly examined for viability by the quantitative viability counting method and direct qualitative outgrowth test for comparison. All testing was done on triplicate pegs in three separate trials (9 replicates in total). Controls were as stated previously.

### Statistical Analysis

For biofilm formation on MBEC pegs, the following statistical analyses were performed as suggested by Harrison et al. [14] including, determination of mean, standard deviation and one-way analysis of variance (ANOVA) comparing the mean viable cell counts of pooled rows within and between plates. These analyses were used to verify that the described method for biofilm inoculation and formation of each test organism produced equivalent biofilm growth on different rows within and between the MBEC plates over the test period of 30 days. For comparison of TBF and BBF and the resultant bioburden following HLD challenge, the Student's t-test (unpaired, two-tailed) was used, as in similar studies [18].

## Results

The survival of a range of representative microorganisms (bacteria, and fungi) in TBF and BBF was evaluated to determine the impact of an organic matrix and disinfectant chemistry. The modelling system produced reproducible results and MBEC statistical analyses showed no significant variation in counts regardless of peg location on or between plates, indicating equivalent biofilm formation. Resulting statistical determinations using ANOVA for each test organism were: *E. faecalis*, p = 0.252; *P. aeruginosa*, p = 0.153; and *C. albicans*, p = 0.893. Validation of the recovery method used to harvest biofilm and BBF from the pegs was performed using a standard live/dead fluorescent staining technique. Microscopic examination of the pegs before and after repeated rounds of harvesting from the pegs indicated that essentially 100% of organisms on the peg were recovered by the first round of harvesting (data not shown).

We specifically chose to evaluate GLUT efficacy using 20 minutes exposure at room temperature rather than using the manufacturer's recommended exposure time of 45 minutes at 25°C. This was done because in clinical settings it is widely accepted that 20 minutes is appropriate for HLD using GLUT. The use of GLUT exposure for 20 minutes exposure at room temperature for HLD is stated in the current APIC guideline as acceptable providing the endoscope has been thoroughly cleaned [12].

**Figure 1:**
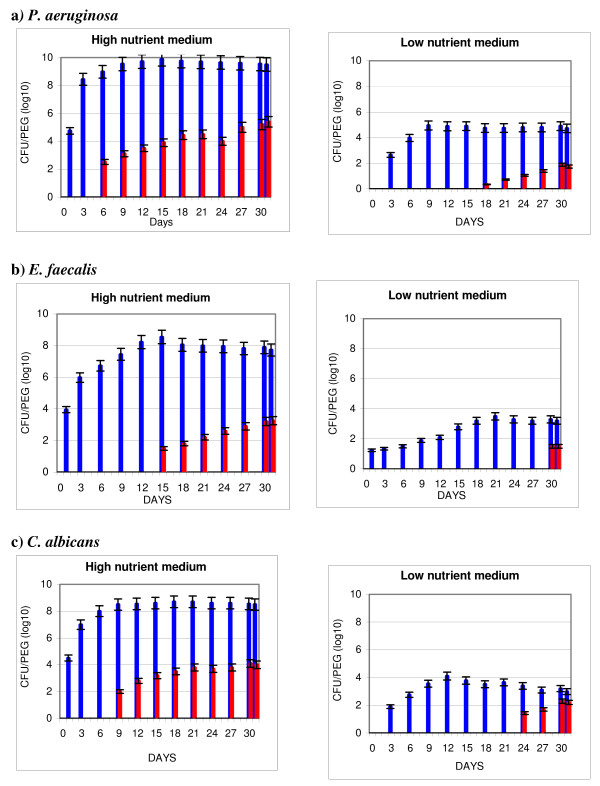
**Traditional biofilm (TBF): impact of Glutaraldehyde on microbial survival**. P. aeruginosa (a), E. faecalis (b), and C. albicans (c), were used to form TBF in high nutrient medium (ATS) or low nutrient medium (tap water). Sample pegs carrying TBF on their surface were removed after varying time periods and the bioburden determined after no treatment (positive control) or after drying followed by treatment with 2.6% Glutaraldehyde for 20 minutes. TBF untreated (blue bar), TBF treated with Glutaraldehyde (red bar).

TBF was formed by allowing pegs to remain hydrated between test periods. The ability of *E. faecalis*, *P. aeruginosa*, and *C. albicans *to form TBF under high or low nutrient conditions and the survivability of these organisms when challenged with HLD using GLUT at various time points over the test period is shown in Figure 1. A significantly higher number of surviving organisms (p < 0.0001) resulted for all test organisms when TBF was formed under high compared to low nutrient medium.

**Figure 2:**
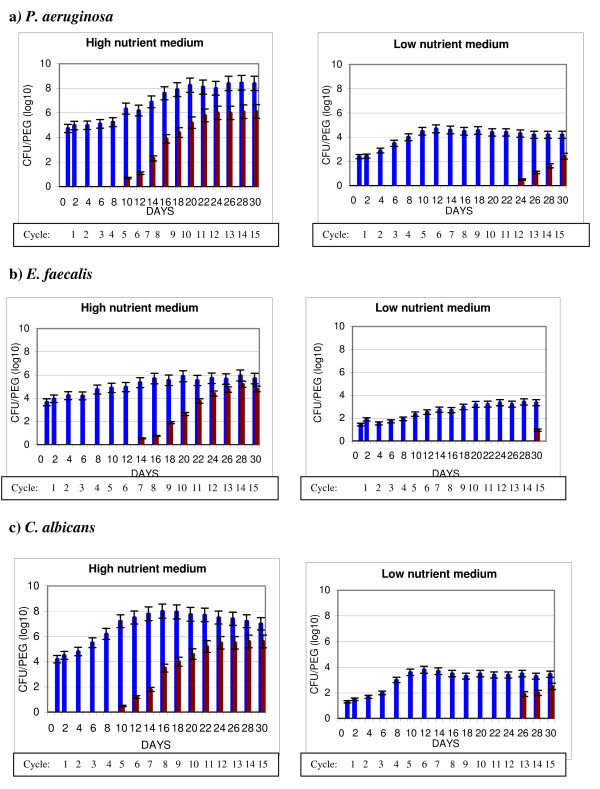
**Buildup biofilm (BBF): impact of Glutaraldehyde on microbial survival**. *P. aeruginosa *(a), *E. faecalis *(b), and *C. albicans *(c), were used to form BBF in high nutrient medium (enzymatic detergent) or low nutrient medium (tap water). Sample pegs carrying BBF on their surface were removed after varying time periods and the bioburden determined after no treatment (positive control) or after treatment with 2.6% Glutaraldehyde for 20 minutes, where every peg with BBF received multiple cycles of treatment as described in Methods. BBF untreated (blue bar), BBF treated with multiple rounds of drying, Glutaraldehyde, and bioburden exposure (red bar).

BBF was formed under conditions of repetitive cycles of drying, HLD and bioburden exposure, mimicking a complete endoscope reprocessing scheme. Two-day cycles were repeated over 30 days with survivability within the BBF examined after each cycle. All data represent BBF seeded in ATS with biofilm developed in either enzymatic detergent or water with cyclic exposure to treatment conditions as stated above. Figure 2 describes survivability of the test organisms in BBF formed under high and low nutrient conditions and challenged with HLD using GLUT at various test times. For all test organisms, a significantly higher number of organisms survived GLUT challenge when BBF was formed under high compared to low nutrient medium (p < 0.0001).

**Figure 3:**
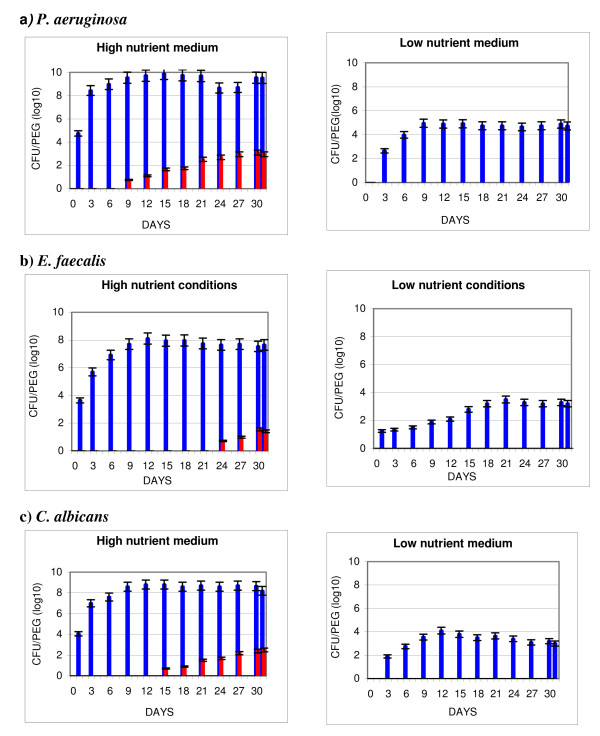
**Traditional biofilm (TBF): impact of Accelerated Hydrogen Peroxide on microbial survival**. *P. aeruginosa *(a), *E. faecalis *(b), and *C. albicans *(c), were used to form TBF in high nutrient medium (ATS) or low nutrient medium (tap water). Sample pegs carrying TBF on their surface were removed after varying time periods and the bioburden determined after no treatment (positive control) or after drying followed by treatment with 7% Accelerated hydrogen peroxide (AHP) for 20 minutes. TBF untreated (blue bar), TBF treated with AHP (red bar).

**Figure 4:**
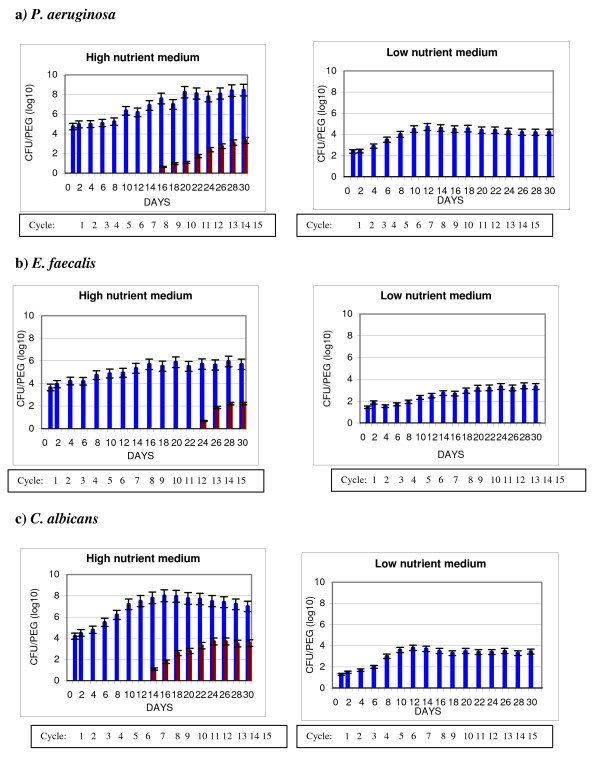
**Buildup biofilm (BBF): impact of Accelerated Hydrogen peroxide on microbial survival**. *P. aeruginosa *(a), *E. faecalis *(b), and *C. albicans *(c), were used to form BBF in high nutrient medium (enzymatic detergent) or low nutrient medium (tap water). Sample pegs carrying BBF on their surface were removed after varying time periods and the bioburden determined after no treatment (positive control) or after treatment with 7% Accelerated hydrogen peroxide (AHP) for 20 minutes, where every peg with BBF received multiple cycles of treatment as described in Methods. BBF untreated (blue bar), BBF treated with multiple rounds of drying, AHP, and bioburden exposure (red bar).

Figures 3 and 4 are similar to Figures 1 and 2 respectively, however HLD is achieved with AHP. All test organisms also showed significantly higher numbers of resulting organisms (p < 0.0001) when either TBF or BBF were formed under high compared to low nutrient conditions.

Additional statistical analyses of maximum resultant microbial loads following HLD challenge demonstrated significant differences (p < 0.0001) for all organisms in the following comparisons: TBF compared to BBF challenged with either GLUT or AHP; GLUT compared to AHP challenge of either BBF or TBF; BBF formed under high nutrient conditions compared to TBF formed under low nutrient conditions, when challenged with GLUT; BBF formed after 10 cycles compared to TBF after a single cycle, with formation under high nutrient conditions and exposure to GLUT.

Table 1 summarizes the breakthrough survival to various disinfectant challenges of test organisms grown under tested conditions for biofilm formation. The time period to detectable survival was similar in TBF (recorded in days) and BBF (recorded in 2-day cycles) for all organisms except *P. aeruginosa*, which required longer times for detectable survival in cyclic BBF than for TBF.

**Table 1:** Summary of Initiation of breakthrough survival in various biofilm formations

		Breakthrough initiation^1^			
**Nutrient** **medium**	**Organism**	**TBF** **Treatment:** **GLUT**	**BBF** **Treatment: GLUT**	**TBF** **Treatment:** **AHP**	**BBF** **Treatment:** **AHP**

High^2^	*E. faecalis*	15	7	24	12

	*P. aeruginosa*	6	5	9	8

	*C. albicans*	9	5	15	7

					

Low^3^	*E. faecalis*	30	15	NBD^4^	NBD

	*P. aeruginosa*	18	12	NBD	NBD

	*C. albicans*	24	13	NBD	NBD

The day (in TBF) or cycle (in BBF) when breakthrough growth was first detected in traditional biofilm or buildup biofilm formed by repetitive exposure to drying, HLD, and reseeding. Results are an average of 9 replicates; SD ≤ 10%.

^1 ^For TBF, the breakthrough time is recorded in days. For BBF, the breakthrough time is recorded in cycles (of a 2-day cycling period).

^2 ^High nutrient media (ATS (for TBF) or Enzymatic detergent (for BBF))

^3 ^Low nutrient media (Water)

^4 ^NBD: no breakthrough detected; limit of detection for viability counting is 10 cfu/peg

Table 2 summarizes the rate of survival in TBF and BBF grown under high nutrient conditions (i.e., ATS for TBF and enzymatic detergent for BBF). Overall BBF demonstrated significantly greater increase in survivability once survivors were detectable particularly following GLUT challenge (p < 0.0001).

**Table 2:** Rate of biofilm survival in TBF^1 ^and BBF^1 ^following HLD with GLUT and AHP

	Rate of survival^2^			
**Organism**	**TBF** **Treatment:** **GLUT**	**BBF** **Treatment: GLUT**	**TBF** **Treatment:** **AHP**	**BBF** **Treatment:** **AHP**

*E. faecalis*	2	10	2.3	3.1

*P. aeruginosa*	2	8.6	3.4	4.9

*C. albicans*	2	9.3	3.4	3.8

^1 ^TBF and BBF grown under high nutrient conditions

^2 ^Rate of survival was calculated by the ratio of:

Maximum Log_10_CFU/Initial breakthrough Log_10_CFU

The qualitative indirect outgrowth testing method was used to establish the detectibility and recoverability of damaged and/or low levels of viable organisms embedded within TBF or BBF. This protocol encouraged the release of embedded organisms by sonication coupled with recovery and growth in enriched media over 30 days. Results, shown in Table 3, detail BBF resulting from repeated cycles of drying and HLD (without re-exposure to bioburden) to assess whether low levels of microbial survival in BBF occurs. Results indicate that despite no detectable viable organisms for the quantitative counts (limit of detection 10 cfu/peg), there can be low- level microbial survival to GLUT in TBF and BBF.

**Table 3:** Survival of organisms embedded in TBF or BBF^1 ^following HLD^2^: indirect qualitative outgrowth testing compared to quantitative viability^3 ^results

Microorganism: BFF	Average cfu/peg^4 ^(Log_10_)	Outgrowth^5^ # Positive/#Tested	Average time to detection (days) for outgrowth
***HLD: 2.6% GLUT:***			

*P. aeruginosa:*			

TBF – Day 3	<LD^6^	3/9	4

TBF – Day 15	3.7	7/9	1

TBF – Day 30	4.8	9/9	1

			

BBF – Day 3	<LD	4/9	20

BBF – Day 15	<LD	2/9	10

BBF – Day 30	1.9	2/9	1

***HLD: 7% AHP:***			

*P. aeruginosa:*			

TBF – Day 3	<LD	0/9	-

TBF – Day 15	1.6	4/9	1

TBF – Day 30	2.5	4/9	1

BBF – Day 3	<LD	0/9	-

BBF – Day 15	<LD	0/9	-

BBF – Day 30	<LD	0/9	-

^1 ^3-day cyclical drying/HLD in ATS medium

^2 ^HLD for cyclic BBF pegs and sample pegs of TBF (formed in ATS)

^3 ^Quantitative viability testing on replicate pegs (without enhanced recovery protocol)

^4 ^Average of 9 pins

^5 ^Indirect qualitative outgrowth (enhanced recovery protocol):

TBF or BBF peg placed in 10%FBS-TSB, shake/sonicate/vortex (as per extraction

method), then continue to incubate at 35°C/30d

^6^LD: limit of detection is 10 cfu/peg for quantitative viability counts

When similarly challenged with AHP HLD, survival was detected in mature TBF for *E. faecalis *and *P. aeruginosa *by indirect qualitative outgrowth and quantitative counts. Similar to the GLUT challenge, as TBF matured, survivability to AHP increased. However, detection of viability following AHP challenge required longer recovery times, occurred on fewer pegs, resulted in lower numbers of survivors compared to GLUT challenge, and AHP survival was detected less frequently in BBF compared to TBF (Table 3).

Overall, the results indicated that the indirect qualitative outgrowth was a more sensitive method to detect the presence of viable organisms than the quantitative viability counting method.

## Discussion

Biofilm formation has been suggested in reprocessed flexible endoscopes in spite of adequate reprocessing, [3,2,11,12,19,20] however the practical effect of such biofilm on reprocessing efficacy and infection transmission is unknown [21]. Our experimental results show that both traditional biofilm formation (TBF) and buildup biofilm formation (BBF) reduced the efficacy of microbial killing by the two HLDs evaluated in this study, which is similar to what has been reported [22] for the effect that TBF has on the killing efficacy of antibiotics. Formation under high nutrient conditions resulted in a higher microbial load in a growing biofilm compared to one formed under low nutrient conditions. Avid biofilm formation, particularly under high nutrient conditions (e.g. with *P. aeruginosa *and *C. albicans*) related to earlier survival to HLD. These findings support clinical data suggesting these organisms are difficult pathogens to eliminate in by the endoscopic disinfection process, [4,21] the importance of reducing organic levels during reprocessing, as well as their strong association with medical implant and dental water unit infections [20,23]. Even when biofilm was formed under low nutrient conditions, if challenged with a cross-linking disinfectant, survival resulted. This further emphasizes that dry storage is imperative to maintain a contaminant free scope after storage and before reuse.

Based on the live/dead fluorescent validation tests that we performed and since we performed the peg harvesting in accordance with published methods using the MBEC peg model [13,14] we have assumed that recovery is 100%.

The results of the TBF and BBF models in this study indicate that HLD is effective at killing bioburden within young biofilm but not mature biofilm and highlight the value in studying biofilm formation in reprocessed scopes over extended periods of time. Our data suggests that deep within the biofilm structure (either TBF or BBF), organisms are protected (likely by ECM and organic material) from the disinfectant challenge, particularly from GLUT challenge. This supports current concerns regarding the exposure of low concentrations or activities of biocides to organisms embedded within biofilm and the selection of tolerant bacteria, [2,24] particularly in BBF. The BBF model demonstrated for the first time that although a longer time was needed for organisms to be detected within the BBF compared to TBF, outgrowth of surviving bioburden in BBF was faster and the ultimate level achieved was greater for BBF. These data provide a possible explanation for published reports describing the persistence of residual levels of organisms in scope channels even when proper reprocessing is followed [11,21].

The data from this study supports the value of using the BBF for research and development of improved endoscope reprocessing methods. For example, although Marion et al. [19] and Vickery et al. [2] have reported that detergents capable of stripping biofilm from the channels of flexible endoscopes are more effective, this conclusion was reached using TBF (formed from a single round of conditions reflecting endoscope usage on carriers or in actual scopes) which may be easier to remove than BBF. Therefore, implications for endoscope reprocessing based on studies of young TBF challenged with a disinfectant may not be indicative of the kill ability of organisms in endoscopes repeatedly used and reprocessed over the life of a scope. Studies of BBF may offer an additional level of stringency and relevance to endoscope reprocessing compared to studies of TBF with a single round testing also in determining the efficacy of anti-biofilm agents (e.g. transition metal catalysts, acylated homoserine lactone molecules or biofilm detaching agents) [19].

An enzymatic detergent's role is to remove patient soil by the "digestive" action of the various enzymes included in the formulation. These formulations do not claim to kill microorganisms. The results of our data demonstrate the ability of organisms to replicate in enzymatic detergent at the manufacturer's recommended use-dilution when held at room temperature overnight (normal contact time recommended by the manufacturer for cleaning is at least 5 minutes). Therefore the practice (although not recommended) of leaving patient-used scopes in enzymatic detergent overnight or over the weekend can serve to increase rather than reduce microbial load and protein buildup, thereby hindering efficacy of the disinfection process. This also supports the need to thoroughly rinse endoscopes after the cleaning phase. Furthermore, it raises the question of whether enzymatic detergents are really the optimal approach or whether other types of detergents (e.g. alkaline or surfactant-associated) could be developed that would be superior and that might have microbial killing ability.

The results of our in vitro model study showed that high-level disinfection using glutaraldehyde was less effective than AHP for killing microorganisms in either TBF or BBF. The benefits of AHP chemistry in bioburden reduction have been cited in published reports [25]. In-use studies of GI endoscope disinfection have shown HLD alternatives to aldehyde chemistry (such as oxidants, e.g. peracetic acid and 13% H2O2 -27% lactic acid; aromatic dialdehydes, e.g. ortho-ophthalaldehyde; and quaternary ammonium iodides, e.g. N-duopropenida) have a superior ability to reduce bacterial loads [26]. As indicated by other researchers similar chemistries warrant further studies [19,26,27]. Our data is the first to demonstrate an oxidant's efficacy in TBF and BBF conditions. For example, breakthrough survival was consistently detected sooner with GLUT than with AHP and the ability of microbes to survive exposure to GLUT occurred irrespective of the organic levels used during biofilm formation. Whereas, AHP consistently eliminated detectable viability in all TBF and BBF formed under low nutrient conditions. Although GLUT has an extremely long global track record as a HLD for reprocessing heat-sensitive medical devices, our study demonstrates for the first time that GLUT is not a very effective method to ensure microorganisms are eradicated. Indeed, after only 10 cycles of BBF formation *P. aeruginosa *consistently survived HLD using GLUT at levels of ~6 Log_10_/peg. The implication of these findings is that organisms remain trapped or embedded due to repetitive GLUT cycles that cross-link organic material forming a protective layer shielding them from the HLD. Once microbial survival within the BBF is established then "grow out" occurs quickly once reintroduced into a moist environment. This scenario supports findings by other researchers describing the kill ability of glutaraldehyde in biofilm but inability to remove bioburden with subsequent impairment of cleaning [18]. However in contrast, the study of TBF and particularly BBF in this research allowed for the detection of viable organisms within this fixed bioburden.

Data from this study highlights the possibility that non-detectibility of viable organisms may not imply efficacy of HLD, particularly in BBF formed by repeated exposure to a cross-linking agent. The determination of the absence of residual microorganisms following reprocessing is dependent on the sensitivity of the detection method [26]. In this in-vitro study using the MBEC peg system for BBF qualitative outgrowth in an enriched medium presented a more sensitive method of bacterial detection, as has been shown in in-use testing of actual endoscopes following cleaning and disinfection [26]. However this study further utilized an indirect qualitative outgrowth method for enhanced microbial recovery of trapped or damaged microorganisms. Another promising method for the measurement of low levels of infectious agents and protein has also been suggested by Lipscomb et al. [28] using differential interference contrast microscopy and the fluorescent agent, SYPRO Ruby.

Data from our study suggest that current guidelines for testing of new liquid chemical disinfectants/sterilants that are based on a single cycle of testing, may not reflect the true efficacy over multiple rounds of reprocessing of a reusable device. The implications are that as flexible endoscopes are repeatedly used and reprocessed, the load of bioburden increases, as does the risk of transmission of pathogens. This study is the first to provide experimental data that may provide an explanation for reported observations indicating that problems with microbial contamination of endoscopes occur after repeated use – not when the scopes are first put into use [22,3]. Our evaluation utilized GLUT exposure conditions of 20 minutes of room temperature to ensure the *in vitro *model being developed reflected what is currently accepted in clinical practice [27,15]. It is possible that exposure using the FDA cleared manufacturer's label claim of 45 minutes at room temperature would show different results for GLUT. Despite this caveat, the data presented in this manuscript support the value of performing similar prospective assessments in patient-used flexible endoscopes to determine the clinical relevance of our findings. Again it should be noted that there are no published clinical reports that directly link increased risk of infection transmission for flexible endoscopes that are reprocessed by glutaraldehyde compared to other HLDs.

## Conclusion

Many models of biofilm exist, but they do not account for the repeated use that is inherent for reusable medical devices. The BBF model described in this study provides a novel approach that closely mimics the conditions and cyclic nature of biofilm formation in patient-used flexible endoscopes. The model included repetitive exposure to organic sources (ATS or enzymatic detergent), bioburden, HLD, rinsing and drying. The BBF model, utilizing repeated cycles of exposure to high levels of organic material containing high loads of microorganisms, more closely replicates endoscope reprocessing; whereas traditional biofilm formation in hydrated, low nutritive conditions does not accurately model what happens in flexible endoscopes that are stored dry. The data presented in this BBF modelling study using the MBEC peg system suggested that early in the life of a reprocessed endoscope, microbial survival may not be detectable (due to low numbers, damage or entrapment of organisms) however over time as scopes are continuously used and reprocessed, rapidly escalating numbers of surviving microorganisms can result when a cross-linking agent such as GLUT is used as the HLD with exposure times of 20 minutes at room temperature.

The novel BBF model developed in this report uses conditions that are more likely to reflect the in-use conditions experienced by flexible endoscopes (i.e. cyclic rounds of exposure that mimics reprocessing stages) than those conditions used for traditional biofilm models (i.e. constant hydration). Our MBEC model of BBF may offer valuable insight that can be used to provide evidence based data that would be instructive in advancing current reprocessing guidelines. Overall, the impact of the data from this research shows for the first time that the survival of a wide range of microorganisms can occur in BBF and that the kinetics and rapid outgrowth is significantly faster compared to TBF, which is much less common in medical devices that are stored under dry conditions. Therefore, the data from our in vitro model suggest that for patient-used endoscopes the chance of organisms surviving device reprocessing and being transmitted to another patient would be greater if BBF has developed within the endoscope channels. Further clinical studies are warranted to further evaluate our findings as there are no publications documenting any increased risk of infection transmission for endoscopes processed using GLUT as the HLD.

## Competing interests

Neither MJA nor RH have any direct or indirect personal financial associations with any company that markets hydrogen peroxide, glutaraldehyde or any other high level disinfectants. No research funds for this study were provided by any company. MJA has undertaken contract research projects (unrelated to the current publication) for a variety of companies including: 3 M, bioMerieux, Olympus, Case Medical, Intelligent Hospital Systems, Johnson & Johnson, STERIS and Virox. No monies from these research contracts go to MJA, they are administered through the St. Boniface Research Centre and are used for research related expenses only. MJA acted as a paid consultant for: preparation of a one time literature review/report for STERIS in 2003 and for providing a one time educational microbiology workshop for 3 M staff in 2006. The page fees for this current article will not be paid for by any industry; they will come from MJA's general research account.

## Authors' contributions

This research was performed by RH as part of her Ph.D. research program and the data included in this manuscript are also presented in her thesis results. RH was responsible for performing the experimental work and was primarily responsible for the writing of the manuscript. MJA conceived the project and provided direction for the experimental testing. MJA also participated in the writing of the manuscript.

## Pre-publication history

The pre-publication history for this paper can be accessed here:

http://www.biomedcentral.com/1471-2334/9/56/prepub
